# Consumer‐Focused Osteoarthritis e‐Learning to Complement Physiotherapy Care in People With Knee Osteoarthritis: Protocol for the PEEKO Randomised Controlled Trial

**DOI:** 10.1002/msc.70156

**Published:** 2025-07-03

**Authors:** Rachel K. Nelligan, Rana S. Hinman, Ben Metcalf, Fiona McManus, Karen E. Lamb, Sophie Heywood, Robyn Gibbes, Kim L. Bennell

**Affiliations:** ^1^ Department of Physiotherapy School of Health Sciences The University of Melbourne Centre for Health Exercise and Sports Medicine Melbourne Australia; ^2^ Melbourne School of Population and Global Health University of Melbourne Centre for Epidemiology and Biostatistics Melbourne Australia; ^3^ Faculty of Medicine University of Melbourne Methods and Implementation Support for Clinical and Health (MISCH) Research Hub Dentistry and Health Sciences Melbourne Australia; ^4^ Physiotherapy Department St Vincent's Hospital Melbourne Melbourne Australia; ^5^ Community‐based Consumer Representative, Person with Knee Osteoarthritis Melbourne Australia

**Keywords:** consumer education, e‐learning, exercise, knee, osteoarthritis, patient education, RCT, trial

## Abstract

**Objective:**

To investigate whether the addition of a consumer e‐learning course to verbal information on OA and a home strengthening exercise programme prescribed by a physiotherapist enhances pain and/or physical function outcomes at 24 weeks in people with knee OA.

Primary outcomes are (i) severity of knee pain while walking (11‐point numeric rating scale) at 24 weeks and (ii) physical function at 24 weeks using the Western Ontario and McMaster Universities Osteoarthritis Index subscale. Secondary outcomes include measures of knee‐related quality of life; sport and recreation; self‐efficacy for exercise; pain self‐efficacy; physical activity levels; OA knowledge; perceptions of OA (illness perceptions); fear of movement; belief in the inevitability of needing a knee joint replacement; global rating of change; satisfaction with treatment; exercise adherence; and use of recommended OA self‐management approaches and oral pain medication.

**Methods:**

Two‐arm parallel‐design superiority randomised controlled trial. One hundred thirty‐six community dwelling Australian adults with clinically diagnosed knee OA will be randomly allocated into one of two groups: (i) verbal OA education and a home strengthening programme (control) or (ii) the same intervention plus access to a freely available 4‐module e‐learning course on OA and its recommended management (e‐learning group). Both groups will receive the same scripted verbal OA information and a lower limb strengthening programme over two 30‐min video consultations with a physiotherapist over six weeks. Those in the e‐learning group will receive access to the e‐learning course from their physiotherapist.

Primary outcomes are (i) severity of knee pain while walking (11‐point numeric rating scale) at 24 weeks and (ii) physical function at 24 weeks using the Western Ontario and McMaster Universities Osteoarthritis Index subscale. Secondary outcomes include measures of knee‐related quality of life; sport and recreation; self‐efficacy for exercise; pain self‐efficacy; physical activity levels; OA knowledge; perceptions of OA (illness perceptions); fear of movement; belief in the inevitability of needing a knee joint replacement; global rating of change; satisfaction with treatment; exercise adherence; and use of recommended OA self‐management approaches and oral pain medication.

**Conclusion:**

This RCT evaluates whether supplementing physiotherapy care with consumer‐focused e‐learning improves outcomes for people with knee OA.

**Abbreviations**
KOOS: Knee Injury and Osteoarthritis Outcome Score
NRS: Numerical Rating Scale
OA: osteoarthritis
RCT: randomised controlled trial
WOMAC: Western Ontario and McMaster Universities Osteoarthritis Index

## 1 Background

Osteoarthritis (OA) is a chronic joint condition affecting 6 million people worldwide (Steinmetz et al. 2023). It typically involves the knee and has a significant impact on those affected, causing joint pain, impaired function and reduced quality of life (Hunter and Bierma‐Zeinstra 2019). In the absence of a known cure, the aim of treatment is to alleviate joint symptoms and prevent negative quality of life impacts with condition‐specific education, exercise and weight management (if indicated) recommended for all people with OA (Bannuru et al. 2019; Moseng et al. 2024; The Royal Australian College of General Practitioners 2018).

Despite the universal recommendation that all people with OA participate in exercise‐based treatment, regardless of symptom severity (Bannuru et al. 2019; Moseng et al. 2024; The Royal Australian College of General Practitioners 2018; Rausch Osthoff et al. 2018; van Doormaal et al. 2020), evidence has shown that exercise has only modest effects on joint pain and physical function (Fransen et al. 2015). Numerous studies have demonstrated that people with OA commonly hold misconceptions about the nature of their condition, its trajectory, and the effect of exercise on their joint health, which can erode their confidence in the safety and efficacy of exercise as a treatment (de Oliveira et al. 2020; Bunzli et al. 2019). Such negative exercise beliefs may lead to poor exercise uptake and reduced exercise adherence and result in low outcome expectations of exercise as a treatment (de Oliveira et al. 2020; Bunzli et al. 2019; O'Brien et al. 2019)—all of which may reduce the ability of exercise treatment to positively impact clinical outcomes.

Physiotherapists, as the primary providers of exercise treatment for knee OA, face various challenges in delivering detailed and accurate consumer education to address OA misconceptions to support exercise prescription. These include limited time, lack of confidence and insufficient expertise (Briggs et al. 2019). Indeed, people with OA have expressed their dissatisfaction with the quality and amount of OA information currently available to them (Wluka et al. 2016). Additionally, many people with OA encounter obstacles in accessing physiotherapy for ongoing care, such as high costs and limited availability (Dobson et al. 2016). Therefore, it is warranted to explore new, scalable and efficient methods for delivering consistent consumer education that supports exercise prescription, especially with minimal clinician contact, to potentially improve exercise outcomes.

To improve consumer access to high‐quality education information about OA and its recommended management, we developed a consumer‐focused e‐learning course informed by educational theory and evidence‐based OA recommendations (Nelligan et al. 2023). The course is freely available worldwide via the online education platform FutureLearn (see https://go.unimelb.edu.au/6ume). In a randomised controlled trial of 120 people, the course was found to improve OA knowledge in people with hip and/or knee OA when compared to a typical OA education intervention: an electronic OA information pamphlet available from a reputable consumer organisation (Nelligan et al. 2025). Building on this work, we now seek to explore whether the course can enhance clinical outcomes from home‐based exercise that is prescribed by physiotherapists in a limited number of consultations.

Specifically, our primary objective is to investigate whether the addition of the e‐learning course to verbal information on OA and its management and a home‐based strengthening exercise programme prescribed over two consultations with a physiotherapist enhances pain and/or physical function outcomes at 24 weeks in people with knee OA. We also aimed to explore whether the programme will improve secondary outcomes at 24 weeks and to identify the proportion of participants achieving minimum clinically important differences in the primary outcomes (experimental group compared to control group).

## 2 Methods

Two arm, parallel group, superiority randomised controlled trial (RCT) conducted at the University of Melbourne (trial sponsor). Reporting of this protocol complies with SPIRIT guidelines (Chan et al. 2015) and TIDieR (Hoffmann et al. 2014). Reporting of the trials findings will comply with CONSORT (Moher et al. 2012). The trial has been prospectively registered with the Australian New Zealand Clinical Trials Registry (ID: ACTRN12624000418572, 5th April 2024).

### 2.1 Participants

Participants will have knee pain that is consistent with a clinical diagnosis of knee OA (NICE 2022) and will meet all other eligibility criteria (see Table 1). Participants will be recruited from the Australian‐wide community via online advertisements (e.g., social media) and our volunteer database. Online forms using REDCap (Research Electronic Data Capture hosted at the University of Melbourne) (Harris et al. 2019, 2009) will be used to obtain informed consent and subsequently baseline questionnaire data from all participants.

**TABLE 1:** Eligibility criteria.

Inclusion criteria	Exclusion criteria
Live in Australia; Aged 45 years or over; Have an unreplaced/native knee joint with: Activity‐related pain at the joint; Joint morning stiffness that lasts ≤ 30 min or no morning stiffness at the joint; History of pain for ≥ 3 months at the joint; and Joint pain on most days of the past month; Report overall average knee pain in the past week during walking ≥ 4 on 11‐point numerical rating scale (NRS; 0 = no pain, 10 = worst pain possible); Willing to participate in video consultations for physiotherapy appointments; Have access to a computer with internet connection and an email address; and Able to give informed consent and willing to commit to all study evaluation and assessment procedures.	Scheduled for or planning lower limb joint surgery in the next 9 months; Previous arthroplasty on the most painful knee; Recent knee injection on the most painful knee (past 6 months); Recent knee surgery on either knee (past 6 months); Consulting/ed physiotherapy or doing regular strengthening (at least once per week) exercise for knee (past 6 months); Self‐reported inflammatory arthritis (e.g., rheumatoid arthritis); Any neurological or other condition affecting lower limbs; Any unstable/uncontrolled cardiovascular condition; Completed an online educational course about OA that involved at least 2 h of learning in total in the past 12 months; and/or Unable to easily read/understand English.

### 2.2 Procedures

**FIGURE 1:**
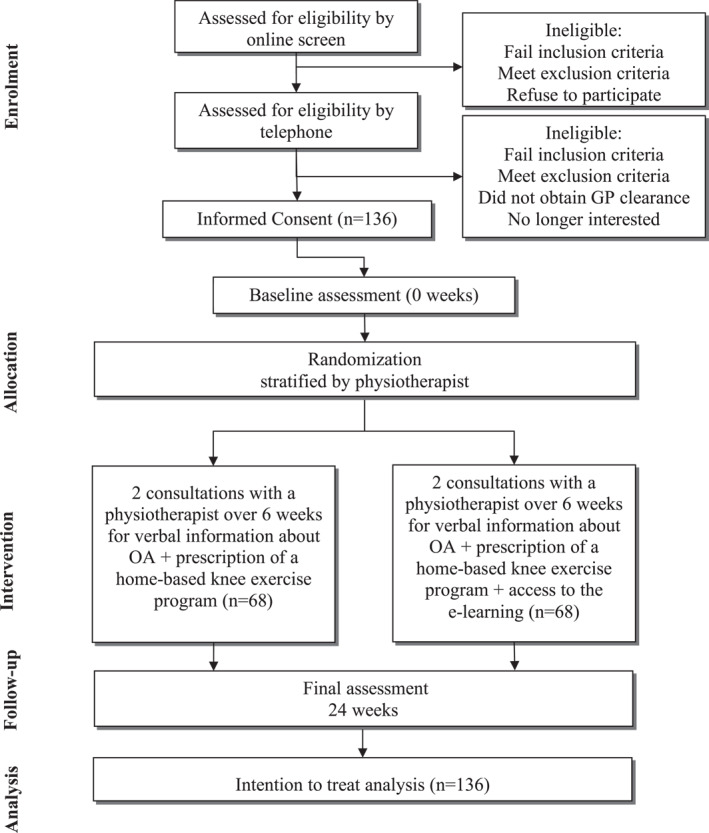
Flow diagram of trial procedures.

Figure 1 outlines trial phases. Participants will first complete an online eligibility survey (REDCap) that presents the study's plain language statement and questions assessing the eligibility criteria (Table 1). Following this, the study coordinator will telephone potentially eligible participants to provide a verbal description of the study, confirm eligibility and confirm participants' willingness to comply with and commit to all trial procedures. Eligible participants will then be emailed a link to access the study's plain language statement, online consent form and baseline questionnaire. On completion of the online consent and baseline questionnaire, participants will be enrolled in the study and allocated a unique study ID code. If a participant has bilaterally eligible joints, the most symptomatic joint will be deemed the study joint. If a participant has bilaterally eligible joints that are equally symptomatic, the participant will be asked to select one joint as the study joint.

### 2.3 Randomisation and Blinding

Participants will be randomised (1:1 ratio) into one of two groups i) e‐learning (experimental group) or ii) no e‐learning (control group), using randomly permuted blocks of varying sizes, stratified by physiotherapist (considering 10 physiotherapists which will account for the 8 study physiotherapists plus an additional 2 to cover any physiotherapists that may drop out of the study). The randomisation list will be computer‐generated by an independent statistician and carried out centrally to ensure concealment. Based on the nature of the interventions (e‐learning or no e‐learning), participants are not considered blinded. However, limited disclosure will be used to blind them to what the intervention/comparator is and to the study hypothesis. During enrolment, participants will be informed that the study is investigating two ways of providing information about OA and its management (verbally only vs. verbal plus an online resource) to see which may best support exercise prescribed by a physiotherapist via video conferencing. They will not be given explicit details about the e‐learning course (experimental intervention). As the primary and secondary outcomes are participant‐reported and participants are not blinded, by default, the assessors of these outcomes are not blinded. As the same physiotherapists will be delivering the interventions in both study arms, physiotherapists will not be blinded. The statistical analysis plan will be written and published on the Centre's website while the biostatisticians are blinded to group allocation, and the main analyses will be performed while the biostatisticians are blinded to group names.

### 2.4 Interventions

- No e‐learning group: verbal OA information and strengthening exercise (control intervention)

Participants in this group will receive two consultations with a physiotherapist over Zoom videoconferencing software (Zoom Video Communications Inc., USA) over 6 weeks. Participants will be seen by the same physiotherapist for both consults (one of eight registered musculoskeletal physiotherapists from across Australia). The first of two physiotherapy consultations will be in approximately week 1 and the second in week 6 (none will be made beyond the end of week 7). The purpose of the consultations will be for the physiotherapist to provide verbal OA information and to prescribe a home‐based strengthening exercise programme. Physiotherapists will consult from their clinic and participants will be based at home or wherever is suitable (e.g., work). Electronic (REDCap) semi‐structured consultation notes will be recorded by the physiotherapist for each consultation. These will be reviewed by the research staff to ensure physiotherapist adherence to trial protocols. Participants will be provided an information booklet for reference about how to prepare for the first consultation and a booklet of strength exercises, as well as 4 coloured resistance exercise bands (red, green, blue, and black) for performing strengthening exercises at home, which they can keep beyond the trial. Those allocated to this group will be provided access to the e‐learning course on completion of their involvement in the study (completed 24‐week outcome measures).

*OA information/education component:* In consultation 1, the physiotherapist will provide verbal information about OA and its recommended management and address any related participant questions. The information will be scripted (script written by the research team in collaboration with the study's eight musculoskeletal physiotherapists) and aims to reflect what may be provided in a real‐world consult. In consult 2, the physiotherapist will allow up to 10 min to address any questions the participant may have about OA and its management. The physiotherapist will answer participant questions based on their clinical knowledge.

*Exercise component (20 min):* In consultation 1, the physiotherapists will prescribe 4 strengthening exercises to be performed at home three times/week, including two quadriceps exercises (from 17 options such as squats and seated knee extensions), one hip (from a list of 13 options such as crab walking or bridges) and one calf (from a list of 4 options including calf raises). The exercises are described in the booklet sent out to participants. The physiotherapist will select the most suitable for the participant based on the participant’s subjective/objective presentation and their goals. The intensity for the strengthening exercises will aim for five to seven out of ten (hard to very hard) on the modified Borg Rating of Perceived Exertion CR‐10 scale (Borg 1998) for strength training. Progression will be in accordance with American College of Sports Medicine guidelines (Liguori 2020) by adjustments to repetitions, direction, and speed of movements, increasing resistance, and/or changing stance surface. Participants will be advised how to independently progress their exercise programme. Physiotherapists will be provided access to a website containing a video library of exercises to allow them to provide real‐time demonstrations of exercises to participants over Zoom. This will be shared with the participants via a share‐screen feature on Zoom so that the participant is able to view a demonstration of the exercise during the consultation. Participants will not have access to the online video library outside the consultations. In consult 2, the physiotherapist will review and modify the home exercise programme to maintain moderate intensity and participants will be further advised how to independently progress their exercise programme in the future. Participants will be encouraged to continue with their strengthening exercises (three times per week) until the final assessment (24‐week). Physiotherapists will record attendance at each session to monitor adherence. Participants will also answer survey questions about how often they completed the exercises at 24 weeks.

- e‐learning group: verbal OA information and strengthening exercise PLUS consumer e‐learning course (experimental intervention)

Participants in this group will receive the same intervention as described for the control group. In addition, participants in this group will be given access to the free e‐learning course, Taking Control of Your Knee and Hip Osteoarthritis, available on the online education platform FutureLearn. In consultation 1, the physiotherapist will use the Zoom ‘share screen’ function to orientate the participant to the e‐learning registration page. Immediately following consult 1, the physiotherapist will email course access details, as well as a Microsoft Word document where participants can record questions to ask the physiotherapist related to the course content at consult 2. Participants will be instructed to complete the course in the coming 4 weeks before consultation 2. As for those in the control group, in consult 2, the physiotherapist will allow up to 10 min to address any questions the participant may have about OA and its management. For participants in this group, in addition to being asked to continue with their strengthening exercises (three times per week) until the final assessment, participants will also be encouraged to trial any other management approaches recommended within the e‐learning course that may interest them.

### 2.5 eLearning Course Content and Development

**FIGURE 2:**
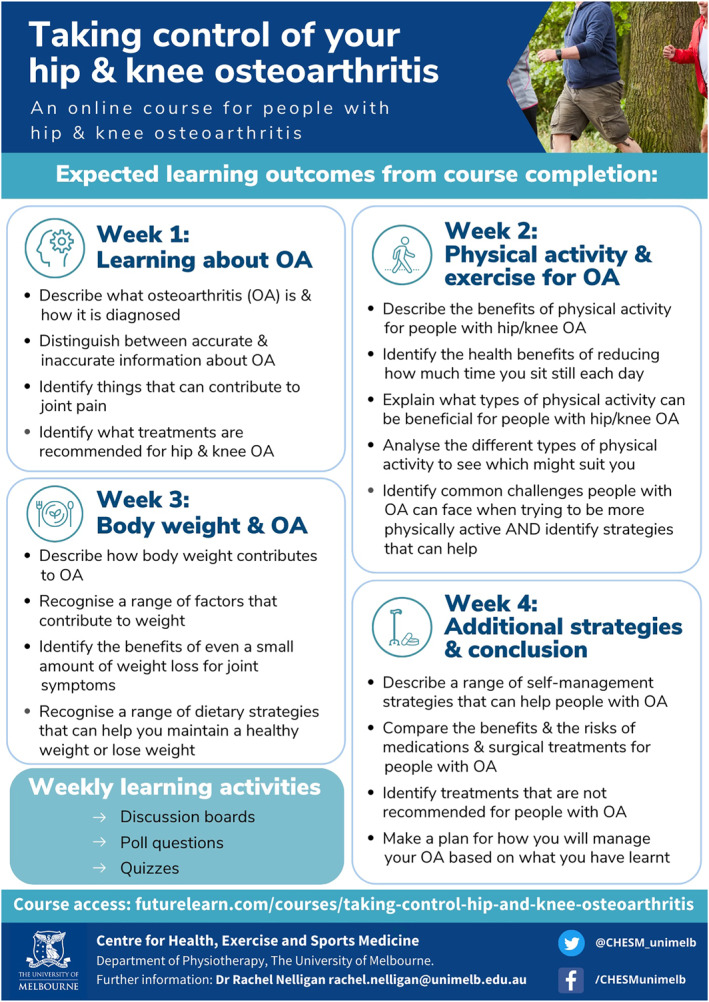
Learning outcomes per module (reproduced from (Nelligan et al. 2023)).

The e‐learning course contains educational information about OA and its management that aligns with best evidence/clinical guideline recommendations for hip/knee OA (French et al. 2015; Hinman et al. 2020; NICE 2022; The Royal Australian College of General Practitioners 2018). Its development and contents are extensively documented elsewhere (Nelligan et al. 2023). Briefly, 348 people with hip/knee OA were involved in the eLearning design via an online survey to gauge interest in the intervention concept (eLearning) and its proposed content. Additionally, a consumer review team (5 people with hip/knee OA) provided feedback (totalling > 21 h) on versions of the eLearning using an iterative think aloud process. The final course includes four modules: (1) learning about osteoarthritis; (2) physical activity and exercise for osteoarthritis; (3) body weight and osteoarthritis; and (4) additional management strategies and conclusions. Information is presented using written text, videos, infographics, downloadable resources (including physical activity/exercise logbook and action plan templates) and learning activities. Each module includes a range of learning activities (e.g., non‐moderated discussion boards to promote learner interaction, poll questions enabling learner comparison, quizzes aligned with learning objectives, action plan templates to facilitate turning learnings into action). The learning outcomes of each module are listed in Figure 2. In total, the time required to complete all four modules and learning activities was approximately four hours (one hour per module).

### 2.6 Physiotherapist Recruitment and Training

Eight physiotherapists will be recruited from the Australia‐wide community. They will be required to work in private practice, have more than 5 years of clinical experience, and have prior training and experience providing knee OA exercise treatment via videoconferencing from their involvement in our other RCTs (Bennell et al. 2022; Hinman et al. 2022). Prior to seeing their first participant, physiotherapists will be required to undertake 3 h of study specific training, led by the chief investigator that will cover the trial protocol and procedures. They will also be required to complete the e‐learning course.

### 2.7 Outcomes

Baseline descriptive data and outcome measures are presented in Table 2. Baseline and 24‐week assessments will be completed remotely via online questionnaires (REDCap). Participants completing 24‐week assessments will be given a $AUD50 gift voucher in gratitude for their participation.

**TABLE 2:** Schedule of enrolment, intervention, and assessments.

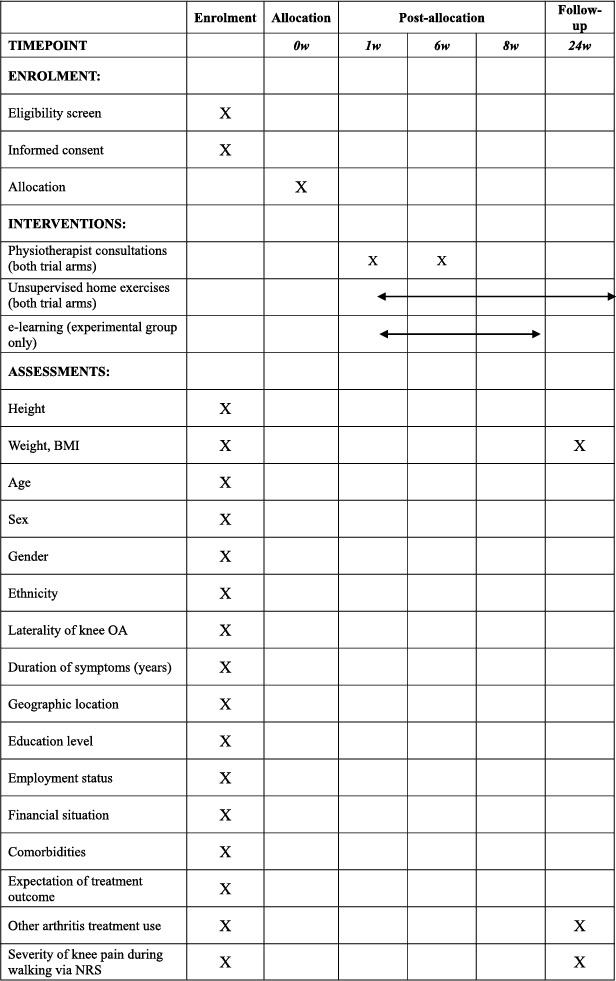 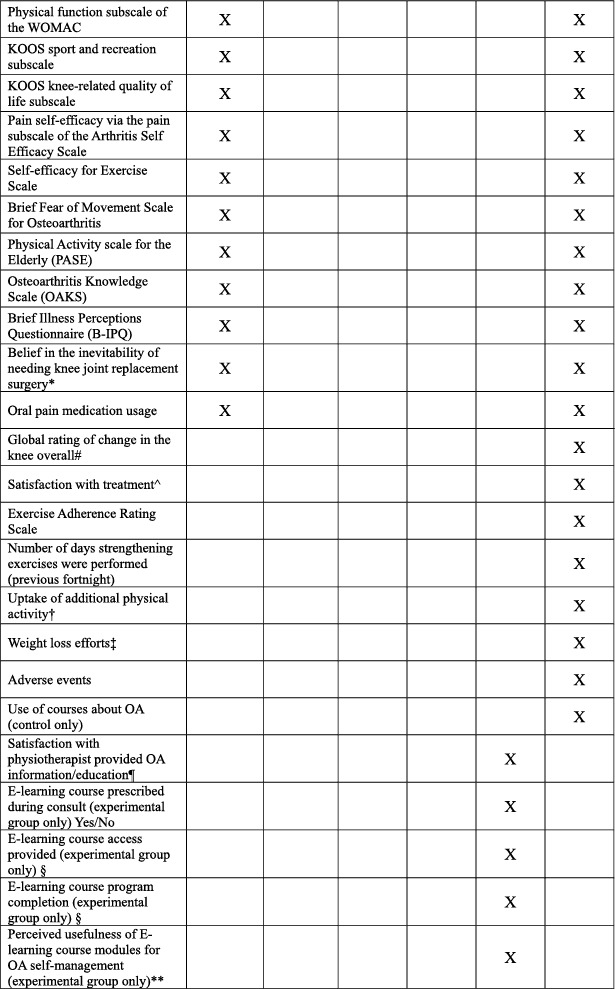 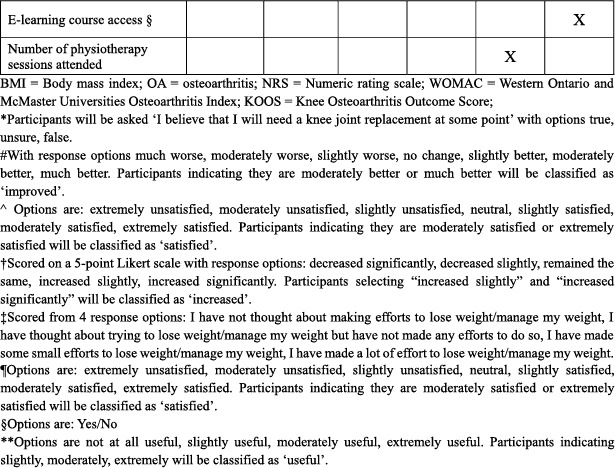

#### 2.7.1 Primary Outcomes

The two self‐reported primary outcomes are psychometrically acceptable, reliable and valid measures recommended for use in clinical trials of knee OA (McAlindon et al. 2015). Both will be collected at baseline and 24 weeks (primary time point).

*Average knee pain during walking in the last week* will be measured using an 11‐point Numerical Rating Scale (NRS) ranging from 0 to 10 (where 0 = no pain; 10 = worst pain possible) (Bellamy 1997).

*Physical function* will be measured using the physical function subscale of the Western Ontario and McMaster Universities Osteoarthritis Index (WOMAC) with responses from 0 to 68, where higher scores indicate greater dysfunction (Bellamy et al. 1988).

#### 2.7.2 Secondary Outcome Measures

A range of self‐reported secondary outcomes will be measured at baseline and 24‐weeks, unless indicated otherwise.

- Sport and recreation subscale of the Knee Injury and Osteoarthritis Outcome Score (KOOS) with 5 items. Scores ranged from 0 to 100 (lower indicating worse function). (Roos et al. 1998)
- Knee‐related quality of life subscale of the KOOS with 4 items. Scores range from 0 to 100 (lower indicating worse quality of life). (Roos et al. 1998)
- The Pain subscale of the Arthritis Self‐Efficacy Scale (ASES pain) with 5 items. Scores range from 1 to 10 (higher indicating greater pain self‐efficacy) (Lorig et al. 1989).
- Self‐efficacy for Exercise (SEE) Scale with 9 items. Scores range from 0 to 90 (higher indicating higher self‐efficacy for exercise) (Resnick and Jenkins 2000).
- Brief Fear of Movement Scale for OA with 6 items. Scores range from 6 to 24 (higher indicating greater fear) (Shelby et al. 2012).
- Physical Activity scale for the Elderly (PASE) with 10 items. Scores range from 0 to 1177 (higher indicating greater levels of physical activity) (Martin et al. 1999).
- Osteoarthritis Knowledge Scale (OAKS) with 11 items. Scores range from 11 to 55 (higher indicating more accurate knowledge about OA) (Darlow et al. 2021).
- Brief Illness Perceptions Questionnaire (B‐IPQ) with 8 items (Broadbent et al. 2006). Scores ranged from 0 to 80 (higher indicating a more threatening view of OA).
- Belief in the inevitability of needing knee joint replacement surgery measured using a study‐specific item. Participants will be asked their level of agreement with the statement ‘I believe that I will need a knee joint replacement at some point’ (response options true, unsure, false will be dichotomised into ‘true’ vs. ‘false or unsure’).
- Global rating of change in the knee overall compared to baseline scored on a 7‐point Likert scale from “much better” to “much worse” (24‐weeks only). Participants indicating they are “moderately better” or “much better” will be classified as ‘improved’.
- Satisfaction with treatment was scored on a 7‐point Likert scale from “extremely unsatisfied” to “extremely satisfied” (24‐weeks only). Participants indicating they are “moderately satisfied” or “extremely satisfied” will be classified as ‘satisfied’.
- Exercise Adherence Rating Scale Section B (Newman‐Beinart et al. 2017) with 6 items (24‐weeks only). Scores ranged from 0 to 24 (higher indicating better adherence).
- Number of days strengthening exercises were performed in the previous fortnight (24‐weeks only). Scores ranged from “0 days” to “14 days”. The outcome will be dichotomised into ‘less than 6 days’ vs. ‘6–14 days’.
- Uptake of physical activity, in addition to physiotherapist prescribed exercise, since baseline with a study specific item: “Apart from the exercises provided by my physiotherapist for this study, in the last 24 WEEKS my physical activity levels have…” with response options on a 5‐point Likert scale from “decreased significantly” to “increased significantly” (24‐weeks only). Participants indicating they have “increased slightly” or “increased significantly” will be classified as ‘increased’.
- Weight loss efforts since baseline (24‐weeks only): “Over the past 24 WEEKS have you made any efforts to lose weight or manage your weight? (e.g., changed your diet)” with 4 response options ranging from “I have not thought about making efforts to lose weight/manage my weight” to “I have made a lot of effort to lose weight/manage my weight”. The number and proportion of each response option will be reported.
- Self‐reported use of oral pain medications for knee pain (24‐weeks only). The number and proportion of participants using common oral pain‐relieving medications for knee pain at least once a week in the prior month will be reported.

#### 2.7.3 Adverse Events

Adverse events will be defined as any untoward medical occurrence in a clinical trial participant that does not necessarily have a causal relationship with the treatment. Adverse events will be ascertained by surveying participants at 24 weeks. Based on this information, the Chief Investigator will determine causality in consultation with other investigators as needed. If the event is related to the trial in the opinion of the Chief Investigator (in consultation with other investigators as required), it will be deemed a related adverse event. A serious adverse event will be defined as any untoward medical occurrence that: (1) results in death; (2) is life‐threatening; (3) requires hospitalisation or prolongation of existing inpatient hospitalisation; (4) results in persistent or significant disability or incapacity; (5) is a congenital anomaly or birth defect; and/or (6) any other important medical condition which, although not included in the above, may require medical or surgical intervention to prevent one of the outcomes listed.

*Process measures:* A range of process measures will also be collected. Table 2 lists all process measures and their assessment timepoints.

### 2.8 Data Analysis, Monitoring and Auditing

#### 2.8.1 Sample Size Calculation

A total sample size of 136 (68 per arm) is required to detect a minimally clinically important difference in the physical function outcome of 6 WOMAC units (Angst et al. 2001) at 24 weeks between the two treatment arms with 80% power and an alpha of 0.025 (alpha of 0.05 split between the two primary outcomes), assuming a correlation of 0.35 between baseline and 24 weeks follow‐up, a standard deviation (SD) of 11 WOMAC units and 15% loss to follow‐up (Bennell et al. 2017; Hinman et al. 2024). This sample size is more than sufficient to detect a minimally clinically important difference of 1.8 NRS units in the NRS pain scale (Bellamy et al. 1992), providing > 98% power when assuming 15% loss to follow‐up, a SD of 2.3 NRS units and a correlation of 0.35 between baseline and 24 weeks follow‐up (Bennell et al. 2017; Hinman et al. 2024).

#### 2.8.2 Data Analysis

The analysis will include all participants according to their randomised allocation (intention‐to‐treat). Multiple imputation will be used as the primary analysis to account for missing data if more than 5% of one or both primary outcomes are missing at 24 weeks. For each primary and continuous secondary outcome, mean between‐group differences at 24 weeks will be estimated from multiple linear regression models. The proportion of participants in each group that show an improvement that reaches or exceeds the minimum clinically important difference in NRS pain (≥ 1.8 units) and WOMAC function (≥ 6 units) will be calculated. For this and each binary outcome, groups will be compared using risk ratios and risk differences estimated from logistic regression models. For the ordinal outcome, weight loss efforts, a proportional odds model will be fitted. All models will include adjustment for baseline values, where relevant, and the stratifying variable of the physiotherapist.

To assess whether the treatment effect differs for different subgroups, exploratory analyses will be undertaken to add interaction terms between the treatment arm and each potential moderator in the models for the primary outcomes. For these analyses, pre‐specified moderators will include baseline levels of OA knowledge (OAKS) (Darlow et al. 2022), pain self‐efficacy (ASES pain) (Lorig et al. 1989), fear of movement (Brief Fear of Movement Scale for Osteoarthritis) (Shelby et al. 2012), and perceptions of OA (illness perceptions) (B‐IPQ) (Broadbent et al. 2006).

#### 2.8.3 Monitoring

The research team will meet fortnightly to review recruitment and monitor trial progress.

### 2.9 Patient and Public Involvement

Three hundred and forty‐eight people with hip/knee OA provided input into the concept (consumer eLearning) and proposed content via an online survey. Five people with OA provided input into course development (using an iterative think aloud process to access a course prototype, totalling > 21 h). One person with knee OA (RG) assisted in developing the RCT and its participant‐facing materials (e.g., plain language statement and consent form) and will assist with finding interpretation. The study's eight musculoskeletal physiotherapists provided input into the OA verbal information script provided during the study consultations.

### 2.10 Dissemination Plans

Findings will be disseminated via conference presentations, journal publications, lay summaries to participants, and our Centre's social media channels and consumer and clinician networks.

## 3 Conclusion

This RCT will determine whether adding an e‐learning course designed specifically for consumers with knee OA—which provides comprehensive information on OA and its management—to standard care (verbal OA information and a home‐based strengthening exercise programme prescribed over two physiotherapy consultations) can improve pain during walking and/or physical function outcomes at 24 weeks. It will also explore whether the e‐learning confers additional benefits for a range of other clinical outcomes including knee‐related quality of life; sport and recreation; self‐efficacy for exercise; pain self‐efficacy; physical activity levels; OA knowledge; perceptions of OA (illness perceptions); fear of movement; belief in the inevitability of needing a knee joint replacement; global rating of change; satisfaction with treatment; exercise adherence; and use of recommended OA self‐management approaches and oral pain medication.

## Author Contributions

RKN, KLB and RSH conceived the idea for the RCT. RKN is leading the study and KLB obtained funding. RKN, KLB, RSH, BM, FM, KEL, SH, and RG designed the study. FM formulated and is responsible for the sample size, and statistical analysis plan under the supervision of KEL. RKN drafted the protocol manuscript. All authors provided input into manuscript preparation and approved the final version.

## Ethics Statement

Institutional Human Ethics Committee approval has been obtained from the University of Melbourne (reference number 2024‐28681‐51186‐3). Informed consent will be obtained from all participants prior to baseline questionnaires using online forms (REDCap, Research Electronic Data Capture, hosted at the University of Melbourne). All methods will be carried out in accordance with the Australian Code for the Responsible Conduct of Research, 2018.

## Conflicts of Interest

The authors declare no conflicts of interest.

## Data Availability

The authors have nothing to report.

## References

[msc70156-bib-0001] Angst, F. , A. Aeschlimann , and G. Stucki . 2001. “Smallest Detectable and Minimal Clinically Important Differences of Rehabilitation Intervention With Their Implications for Required Sample Sizes Using WOMAC and SF‐36 Quality of Life Measurement Instruments in Patients With Osteoarthritis of the Lower Extremities.” Arthritis & Rheumatism 45, no. 4: 384–391. 10.1002/1529-0131(200108)45:4<384::Aid-art352>3.0.Co;2-0. 11501727

[msc70156-bib-0002] Bannuru, R. R. , M. C. Osani , E. E. Vaysbrot , et al. 2019. “OARSI Guidelines for the Non‐surgical Management of Knee, Hip, and Polyarticular Osteoarthritis.” Osteoarthritis and Cartilage 27, no. 11: 1578–1589. 10.1016/j.joca.2019.06.011. 31278997

[msc70156-bib-0003] Bellamy, N. , W. W. Buchanan , C. H. Goldsmith , J. Campbell , and L. W. Stitt . 1988. “Validation Study of WOMAC: A Health Status Instrument for Measuring Clinically Important Patient Relevant Outcomes to Antirheumatic Drug Therapy in Patients With Osteoarthritis of the Hip or Knee.” Journal of Rheumatology 15, no. 12: 1833–1840. 3068365

[msc70156-bib-0004] Bellamy, N. , S. Carette , P. Ford , et al. 1992. “Osteoarthritis Antirheumatic Drug Trials. II. Tables for Calculating Sample Size for Clinical Trials.” Journal of Rheumatology 19, no. 3: 444–450. 1578461

[msc70156-bib-0005] Bellamy, N. 1997. “Osteoarthritis Clinical Trials: Candidate Variables and Clinimetric Properties.” Journal of Rheumatology 24, no. 4: 768–778. 9101516

[msc70156-bib-0006] Bennell, K. L. , R. Nelligan , F. Dobson , et al. 2017. “Effectiveness of an Internet‐Delivered Exercise and Pain‐Coping Skills Training Intervention for Persons With Chronic Knee Pain: A Randomized Trial.” Annals of Internal Medicine 166, no. 7: 453–462. 10.7326/m16-1714. 28241215

[msc70156-bib-0007] Bennell, K. L. , S. E. Jones , R. S. Hinman , et al. 2022. “Effectiveness of a Telehealth Physiotherapist‐Delivered Intensive Dietary Weight Loss Program Combined With Exercise in People With Knee Osteoarthritis and Overweight or Obesity: Study Protocol for the POWER Randomized Controlled Trial.” BMC Musculoskeletal Disorders 23, no. 1: 733. 10.1186/s12891-022-05685-z. 35907828 PMC9338658

[msc70156-bib-0008] Borg, G. 1998. Borg's Perceived Exertion and Pain Scales. ISBN: 0880116234. Human Kinetics.

[msc70156-bib-0009] Briggs, A. M. , E. Houlding , R. S. Hinman , et al. 2019. “Health Professionals and Students Encounter Multi‐Level Barriers to Implementing High‐Value Osteoarthritis Care: A Multi‐National Study.” Osteoarthritis and Cartilage 27, no. 5: 788–804. 10.1016/j.joca.2018.12.024. 30668988

[msc70156-bib-0010] Broadbent, E. , K. J. Petrie , J. Main , and J. Weinman . 2006. “The Brief Illness Perception Questionnaire.” Journal of Psychosomatic Research 60, no. 6: 631–637. 10.1016/j.jpsychores.2005.10.020. 16731240

[msc70156-bib-0011] Bunzli, S. , P. O'Brien , D. Ayton , et al. 2019. “Misconceptions and the Acceptance of Evidence‐Based Nonsurgical Interventions for Knee Osteoarthritis. A Qualitative Study.” Clinical Orthopaedics and Related Research 477, no. 9: 1975–1983. 10.1097/corr.0000000000000784. 31192807 PMC7000096

[msc70156-bib-0012] Chan, A. W. , J. M. Tetzlaff , D. G. Altman , et al. 2015. “SPIRIT 2013 Statement: Defining Standard Protocol Items for Clinical Trials.” Revista Panamericana de Salud Públic 38, no. 6: 506–514. PMC5114122 27440100

[msc70156-bib-0013] Darlow, B. , H. Abbott , K. Bennell , et al. 2021. “Knowledge About Osteoarthritis: Development of the Hip and Knee Osteoarthritis Knowledge Scales and Protocol for Testing Their Measurement Properties.” Osteoarthritis and Cartilage Open 3, no. 2: 100160. 10.1016/j.ocarto.2021.100160. 36474995 PMC9718068

[msc70156-bib-0014] Darlow, B. , C. Krägeloh , J. H. Abbott , et al. 2022. “The Osteoarthritis Knowledge Scale.” Musculoskeletal Care 21, no. 2: 516–526. 10.1002/msc.1727. 36573463

[msc70156-bib-0015] de Oliveira, B. I. R. , A. J. Smith , P. P. B. O'Sullivan , et al. 2020. “'My Hip Is Damaged': A Qualitative Investigation of People Seeking Care for Persistent Hip Pain.” British Journal of Sports Medicine 54, no. 14: 858–865. 10.1136/bjsports-2019-101281. 31980419

[msc70156-bib-0016] Dobson, F. , K. L. Bennell , S. D. French , et al. 2016. “Barriers and Facilitators to Exercise Participation in People With Hip And/or Knee Osteoarthritis: Synthesis of the Literature Using Behavior Change Theory.” American Journal of Physical Medicine & Rehabilitation 95, no. 5: 372–389. 10.1097/phm.0000000000000448. 26945211

[msc70156-bib-0017] Fransen, M. , S. McConnell , A. R. Harmer , M. Van der Esch , M. Simic , and K. L. Bennell . 2015. “Exercise for Osteoarthritis of the Knee: A Cochrane Systematic Review.” British Journal of Sports Medicine 49, no. 24: 1554–1557. 10.1136/bjsports-2015-095424. 26405113

[msc70156-bib-0018] French, S. D. , K. L. Bennell , P. J. A. Nicolson , P. W. Hodges , F. L. Dobson , and R. S. Hinman . 2015. “What Do People With Knee or Hip Osteoarthritis Need to Know? an International Consensus List of Essential Statements for Osteoarthritis.” Arthritis Care & Research 67, no. 6: 809–816. 10.1002/acr.22518. 25418120

[msc70156-bib-0019] Harris, P. A. , R. Taylor , R. Thielke , J. Payne , N. Gonzalez , and J. G. Conde . 2009. “Research Electronic Data Capture (REDCap)‐‐A Metadata‐Driven Methodology and Workflow Process for Providing Translational Research Informatics Support.” Journal of Biomedical Informatics 42, no. 2: 377–381. 10.1016/j.jbi.2008.08.010. 18929686 PMC2700030

[msc70156-bib-0020] Harris, P. A. , R. Taylor , B. L. Minor , et al. 2019. “The REDCap Consortium: Building an International Community of Software Platform Partners.” Journal of Biomedical Informatics 95: 103208. 10.1016/j.jbi.2019.103208. 31078660 PMC7254481

[msc70156-bib-0021] Hinman, R. S. , K. D. Allen , K. L. Bennell , et al. 2020. “Development of a Core Capability Framework for Qualified Health Professionals to Optimise Care for People With Osteoarthritis: An OARSI Initiative.” Osteoarthritis and Cartilage 28, no. 2: 154–166. 10.1016/j.joca.2019.12.001. 31838047

[msc70156-bib-0022] Hinman, R. S. , R. K. Nelligan , P. K. Campbell , et al. 2022. “Exercise Adherence Mobile App for Knee Osteoarthritis: Protocol for the MappKO Randomised Controlled Trial.” BMC Musculoskeletal Disorders 23, no. 1: 874. 10.1186/s12891-022-05816-6. 36127677 PMC9487056

[msc70156-bib-0023] Hinman, R. S. , Campbell, P. K. , Kimp, A. J. , et al. 2024. “Telerehabilitation Consultations With a Physiotherapist for Chronic Knee Pain Versus In‐Person Consultations in Australia: the PEAK Non‐Inferiority Randomised Controlled Trial.” Lancet 403, no. 10433: 1267–1278. 10.1016/S0140-6736(23)02630-2. PMID: 38461844. 38461844

[msc70156-bib-0024] Hoffmann, T. C. , P. P. Glasziou , I. Boutron , et al. 2014. “Better Reporting of Interventions: Template for Intervention Description and Replication (TIDieR) Checklist and Guide.” BMJ 348, no. 3: g1687. 10.1136/bmj.g1687. 24609605

[msc70156-bib-0025] Hunter, D. J. , and S. Bierma‐Zeinstra . 2019. “Osteoarthritis.” Lancet 393, no. 10182: 1745–1759. 10.1016/S0140-6736(19)30417-9. 31034380

[msc70156-bib-0026] Liguori, G. 2020. American College of Sports Medicine Guidelines for Exercise Testing and Prescription. Lippincott Williams & Wilkins.

[msc70156-bib-0027] Lorig, K. , R. L. Chastain , E. Ung , S. Shoor , and H. R. Holman . 1989. “Development and Evaluation of a Scale to Measure Perceived Self‐Efficacy in People With Arthritis.” Arthritis & Rheumatism 32, no. 1: 37–44. 10.1002/anr.1780320107. 2912463

[msc70156-bib-0028] Martin, K. A. , W. J. Rejeski , M. E. Miller , M. K. James , W. H. Ettinger Jr. , and S. P. Messier . 1999. “Validation of the PASE in Older Adults With Knee Pain and Physical Disability.” Medicine & Science in Sports & Exercise 31, no. 5: 627–633. 10.1097/00005768-199905000-00001. 10331879

[msc70156-bib-0029] McAlindon, T. E. , J. B. Driban , Y. Henrotin , et al. 2015. “OARSI Clinical Trials Recommendations: Design, Conduct, and Reporting of Clinical Trials for Knee Osteoarthritis.” Osteoarthritis and Cartilage 23, no. 5: 747–760. 10.1016/j.joca.2015.03.005. 25952346

[msc70156-bib-0030] Moher, D. , S. Hopewell , K. F. Schulz , et al. 2012. “CONSORT 2010 Explanation and Elaboration: Updated Guidelines for Reporting Parallel Group Randomised Trials.” International Journal of Surgery 10, no. 1: 28–55. 10.1016/j.ijsu.2011.10.001. 22036893

[msc70156-bib-0031] Moseng, T. , T. P. M. Vliet Vlieland , S. Battista , et al. 2024. “EULAR Recommendations for the Non‐Pharmacological Core Management of Hip and Knee Osteoarthritis: 2023 Update.” Annals of the Rheumatic Diseases 83, no. 6: 730–740. 10.1136/ard-2023-225041. 38212040 PMC11103326

[msc70156-bib-0032] Nelligan, R. K. , R. S. Hinman , T. Egerton , et al. 2023. “Effects of a Massive Open Online Course on Osteoarthritis Knowledge and Pain Self‐Efficacy in People With Hip and/or Knee Osteoarthritis: Protocol for the MOOC‐OA Randomised Controlled Trial.” BMC Musculoskeletal Disorders 24, no. 1: 381. 10.1186/s12891-023-06467-x. 37189094 PMC10184332

[msc70156-bib-0033] Nelligan, R. K. , R. S. Hinman , F. McManus , et al. 2025. “Effects of an eLearning Course for Patients on Osteoarthritis Knowledge and Pain Self‐Efficacy in People With Hip and/or Knee Osteoarthritis: A Randomised Controlled Trial.” Patient Education and Counseling 137: 108792. 10.1016/j.pec.2025.108792. 40300349

[msc70156-bib-0034] Newman‐Beinart, N. A. , S. Norton , D. Dowling , et al. 2017. “The Development and Initial Psychometric Evaluation of a Measure Assessing Adherence to Prescribed Exercise: The Exercise Adherence Rating Scale (EARS).” Physiotherapy 103, no. 2: 180–185. 10.1016/j.physio.2016.11.001. 27913064

[msc70156-bib-0035] NICE . 2022. Osteoarthritis in over 16s: Diagnosis and Management. NICE guideline [NG226]. 36745715

[msc70156-bib-0036] O'Brien, P. , S. Bunzli , D. Ayton , M. M. Dowsey , J. Gunn , and J. A. Manski‐Nankervis . 2019. “What are the Patient Factors That Impact on Decisions to Progress to Total Knee Replacement? A Qualitative Study Involving Patients With Knee Osteoarthritis.” BMJ Open 9, no. 9: e031310. 10.1136/bmjopen-2019-031310. PMC6773346 31551388

[msc70156-bib-0037] Rausch Osthoff, A.‐K. , K. Niedermann , J. Braun , et al. 2018. “2018 EULAR Recommendations for Physical Activity in People With Inflammatory Arthritis and Osteoarthritis.” Annals of the Rheumatic Diseases 77, no. 9: 1251–1260. 10.1136/annrheumdis-2018-213585. 29997112

[msc70156-bib-0038] Resnick, B. , and L. S. Jenkins . 2000. “Testing the Reliability and Validity of the Self‐Efficacy for Exercise Scale.” Nursing Research 49, no. 3: 154–159. 10.1097/00006199-200005000-00007. 10882320

[msc70156-bib-0039] Roos, E. M. , H. P. Roos , L. S. Lohmander , C. Ekdahl , and B. D. Beynnon . 1998. “Knee Injury and Osteoarthritis Outcome Score (KOOS)‐‐Development of a Self‐Administered Outcome Measure.” Journal of Orthopaedic & Sports Physical Therapy 28, no. 2: 88–96. 10.2519/jospt.1998.28.2.88. 9699158

[msc70156-bib-0040] Shelby, R. A. , T. J. Somers , F. J. Keefe , et al. 2012. “Brief Fear of Movement Scale for Osteoarthritis.” Arthritis Care & Research 64, no. 6: 862–871. 10.1002/acr.21626. 22290689 PMC3357444

[msc70156-bib-0041] Steinmetz, J. D. , G. T. Culbreth , L. M. Haile , et al. 2023. “Global, Regional, and National Burden of Osteoarthritis, 1990–2020 and Projections to 2050: A Systematic Analysis for the Global Burden of Disease Study 2021.” Lancet Rheumatology 5, no. 9: e508–e522. 10.1016/S2665-9913(23)00163-7. 37675071 PMC10477960

[msc70156-bib-0042] The Royal Australian College of General Practitioners . 2018. Guideline for the Management of Knee and Hip Osteoarthritis.

[msc70156-bib-0043] van Doormaal, M. C. M. , G. A. Meerhoff , T. P. M. Vliet Vlieland , and W. F. Peter . 2020. “A Clinical Practice Guideline for Physical Therapy in Patients With Hip or Knee Osteoarthritis.” Musculoskeletal Care 18, no. 4: 575–595. 10.1002/msc.1492. 32643252

[msc70156-bib-0044] Wluka, A. E. , L. Chou , A. M. Briggs , and F. M. Cicuttini . 2016. “Understanding the Needs of Consumers With Musculoskeletal Conditions: Consumers’ Perceived Needs of Health Information, Health Services and Other Non‐Medical Services: A Systematic Scoping Review.” Move Muscle, Bone & Joint Health.

